# Treatment of tumor-associated macrophages with PD-1 monoclonal antibodies affects vascular generation in cervical cancer via the PD-1/IRE1α/SHP2/HIF1α signaling pathway

**DOI:** 10.18632/aging.206090

**Published:** 2024-08-28

**Authors:** Xiaohui Hao, Weiwei Zhao, Xianyu Zhang, Xiurong Lu, Cong Wang, Zhilin Zhang

**Affiliations:** 1 Department of Radiotherapy, The First Affiliated Hospital of Hebei North University, Zhangjiakou, Hebei, China

**Keywords:** cervical cancer, tumor-associated macrophages, Sindilizumab, IRE1α, SHP2, HIF1α

## Abstract

Objective: To investigate the effect of PD-1 monoclonal antibodies in tumor-associated macrophages on angiogenesis in cervical cancer and its mechanism of action.

Methods: The effect of PD-1 monoclonal antibodies on the progression of cervical cancer was assessed using the nude mouse xenograft model and HE staining; the impact of PD-1 monoclonal antibodies on cervical cancer cell migration was evaluated using wound healing assay and Transwell assay; the effect on vascular formation in cervical cancer cells was examined using an angiogenesis assay; the impact on the expression of related proteins was tested using Western blotting.

Results: PD-1 monoclonal antibodies in tumor-associated macrophages can regulate and thus inhibit the progression of cervical cancer while promoting the expression of SHP2. Additionally, Sindilizumab inhibited the expression of tissue-type fibrinogen activator K and HIF1α through the PD-1/IRE1α/SHP2 signaling pathway, which inhibited the migration and neovascularization of cervical cancer cells.

Conclusions: This study discovered that PD-1 monoclonal antibodies in tumor-associated macrophages inhibit vascular generation inside cervical cancer by affecting the PD-1/IRE1α/SHP2/HIF1α signaling pathway, providing a new therapeutic target for the treatment of cervical cancer.

## INTRODUCTION

Cervical carcinoma is globally recognized as the second most prevalent cancer among women, marked by the highest mortality rate among all female cancers. Each year, around 500,000 new cases of cervical cancer emerge, with 80% occurring in developing countries. The highest incidence rates are noted in Africa, Central and South America, and Asia [1, 2]. Annually, cervical cancer is responsible for approximately 250,000 deaths worldwide. The prognosis for patients with cervical cancer is closely tied to the stage at which the disease is diagnosed: early-stage diagnosis yields survival rates over 90%, whereas survival rates for those diagnosed in mid-to-late stages are approximately 10%. Thus, the identification of effective therapeutic targets for cervical cancer is crucial [3, 4].

Macrophages, highly adaptable immune cells, excel in engulfing bacteria, viruses, and necrotic or apoptotic cells, as well as other particulates. They play a crucial role in pathogen defense, driving inflammatory responses, and facilitating wound healing, tissue regeneration, and immune regulation. Studies have demonstrated that the tumor microenvironment acts as the primary site for neoplastic cell proliferation and differentiation, with cancer cell metastasis influenced by pro-inflammatory cytokines secreted by tumor-associated macrophages (TAMs). This environment is abundant with inflammatory cells; the complex interactions among these cells perpetuate a continuous inflammatory state. TAMs are instrumental within this array of inflammatory cells, playing an essential role in tumor-associated inflammation. By secreting various anti-inflammatory cytokines, TAMs mitigate immune system activation while promoting neoplastic cell growth and angiogenesis, thereby aiding tumor cell invasion and metastasis [5–7].

Programmed cell death protein 1 (PD-1) is a protein integral to the tumor immune surveillance apparatus of the human immune system. As a member of the immune checkpoint receptors group, it is typically found on activated T cells. Its main role is to preserve the immune system's tolerance to the body's own cells, preventing an auto-immune response. In certain cancers, including cervical cancer, neoplastic cells may express the ligand PD-L1 (Programmed death-ligand 1), which, upon binding to PD-1, effectively "switches off" the T cell-mediated immune response. This allows cancer cells to escape immune detection and augments their proliferation and survival. Furthermore, the interaction between PD-1 and PD-L1 extends to TAMs in the cervical cancer microenvironment, wherein PD-1's pathway activation may shift TAMs from an anti-tumor M1 phenotype to a pro-tumor M2 phenotype, thereby allowing cancer cells to evade immune detection by expressing PD-L1, thereby muting the immune responses of both TAMs and T cells and promoting tumor progression and dissemination [8–10]. In the tumor microenvironment of cervical cancer, TAMs may express PD-1 while cervical cancer cells may express PD-L1. When the PD-1 pathway is activated in TAMs, they may transition from anti-tumor M1-type macrophages to M2-type macrophages that promote tumor growth. Cervical cancer cells evade the immune system's surveillance by expressing PD-L1, inhibiting the immune response of TAMs and T cells, leading to tumor growth and metastasis [11, 12].

In the realm of oncology, the Src Homology 2 domain-containing phosphatase 2 (SHP2) is frequently researched as a crucial facilitator within the PD-1/PD-L1 immune checkpoint signaling framework, playing a significant role in myriad cellular pathways, including those involved in cellular proliferation, differentiation, and survival [13, 14]. Therefore, in this study, we aimed to investigate the effect of PD-1 monoclonal antibodies on cervical cancer and its mechanism of action. We found that PD-1 monoclonal antibodies can influence the expression of the PD-1/IRE1α/SHP2/HIF1α signaling pathway in TAMs and inhibit the migration and neovascularization of cervical cancer cells.

## RESULTS

### Sindilizumab in TAMs inhibits the progression of cervical cancer by promoting SHP2

**Figure 1:**
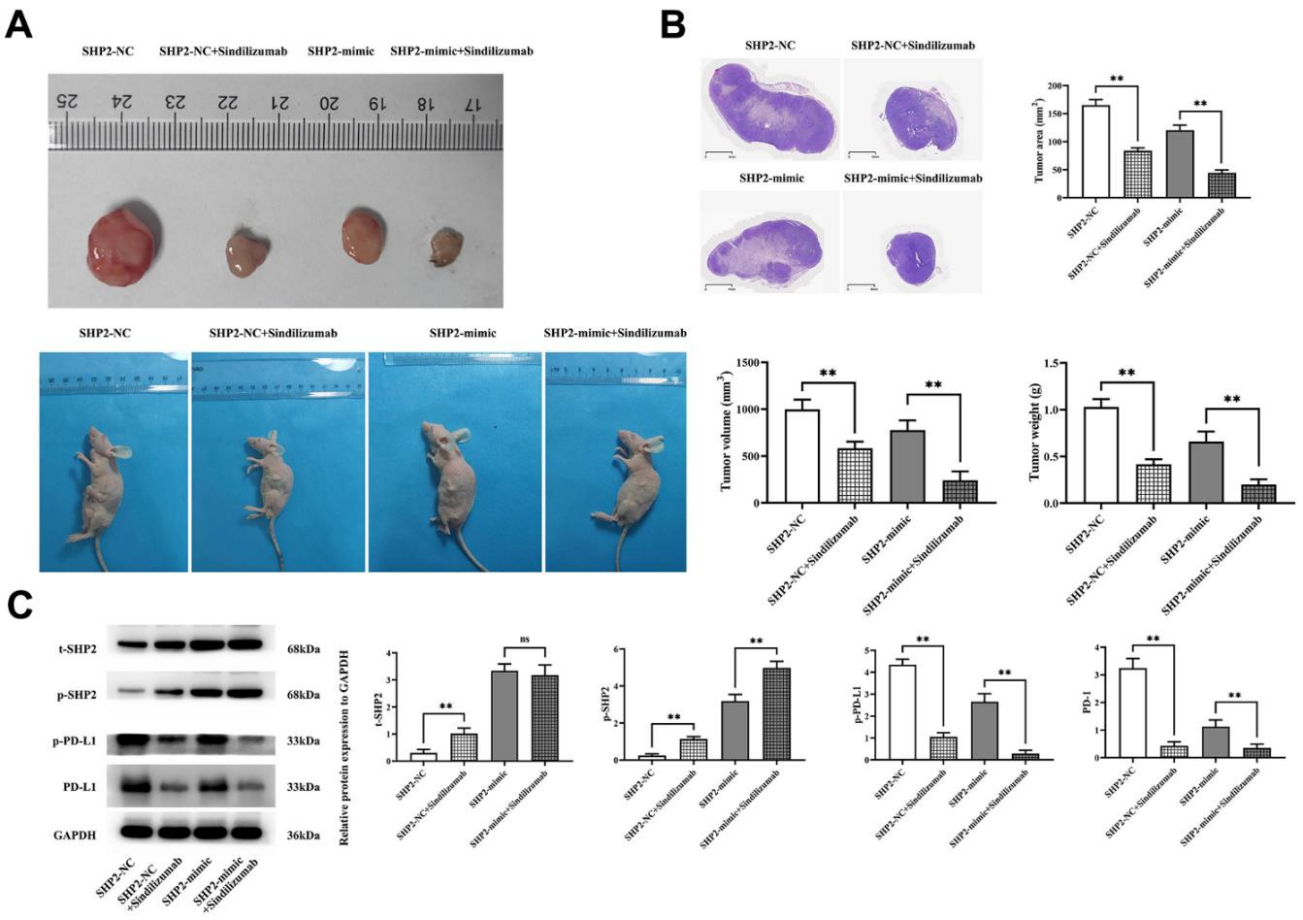
**Impact of PD-1 monoclonal antibodies in TAMs on the progression of cervical cancer.** (**A**) Graphical representation of tumor-bearing nude mice experimental results and statistical graph of tumor volume and weight; (**B**) H&E staining results graph and statistical graph of tumor slice area; (**C**) Western blot bands of t-SHP2, p-SHP2, PD-1, and PD-L1 and relative protein expression levels. GAPDH as a control protein. Data were expressed as mean±SD. **P<0.01; nsP>0.05.

First, we designed an *in vivo* experiment to explore the effect of Sindilizumab on the progression of cervical cancer in TAMs. The tumor-bearing nude mouse experiment showed that tumor volumes in both SHP2-NC and SHP2-mimic groups significantly decreased after the addition of Sindilizumab. Compared to the SHP2-NC group, the SHP2-mimic group showed a significant reduction in tumor volume and weight; compared to the SHP2-NC+Sindilizumab group, the SHP2-mimic+Sindilizumab group had a significantly reduced tumor volume and weight. HE staining results indicated a significant reduction in tumor cross-sectional area for both SHP2-NC and SHP2-mimic groups after treatment with Sindilizumab. Compared to the SHP2-NC group, the SHP2-mimic group displayed a significant reduction in tumor cross-sectional area; compared to the SHP2-NC+Sindilizumab group, the SHP2-mimic+Sindilizumab group had a significantly reduced tumor cross-sectional area. Western blotting was used to detect the expression of related proteins in cancer tissues. The results show that, compared to the SHP2-NC group, the relative protein expression levels of t-SHP2 and p-SHP2 in the SHP2-mimic group were significantly increased, while the relative expression levels of PD-1 and PD-L1 were significantly decreased; compared to the SHP2-NC+Sindilizumab group, the SHP2-mimic+Sindilizumab group showed significantly increased levels of t-SHP2 and p-SHP2, and significantly decreased levels of PD-L1, with no significant difference in PD-1 expression. Compared to the SHP2-NC group, the SHP2-NC+Sindilizumab group showed significantly increased levels of t-SHP2 and p-SHP2, and significantly decreased levels of PD-1 and PD-L1; compared to the SHP2-mimic group, the SHP2-mimic+Sindilizumab group showed significantly increased levels of p-SHP2 and significantly decreased levels of PD-1 and PD-L1, with no significant difference in t-SHP2 expression (Figure 1).

### Sindilizumab in TAMs can regulate the PD-1/IRE1α/SHP2 signaling pathway

**Figure 2:**
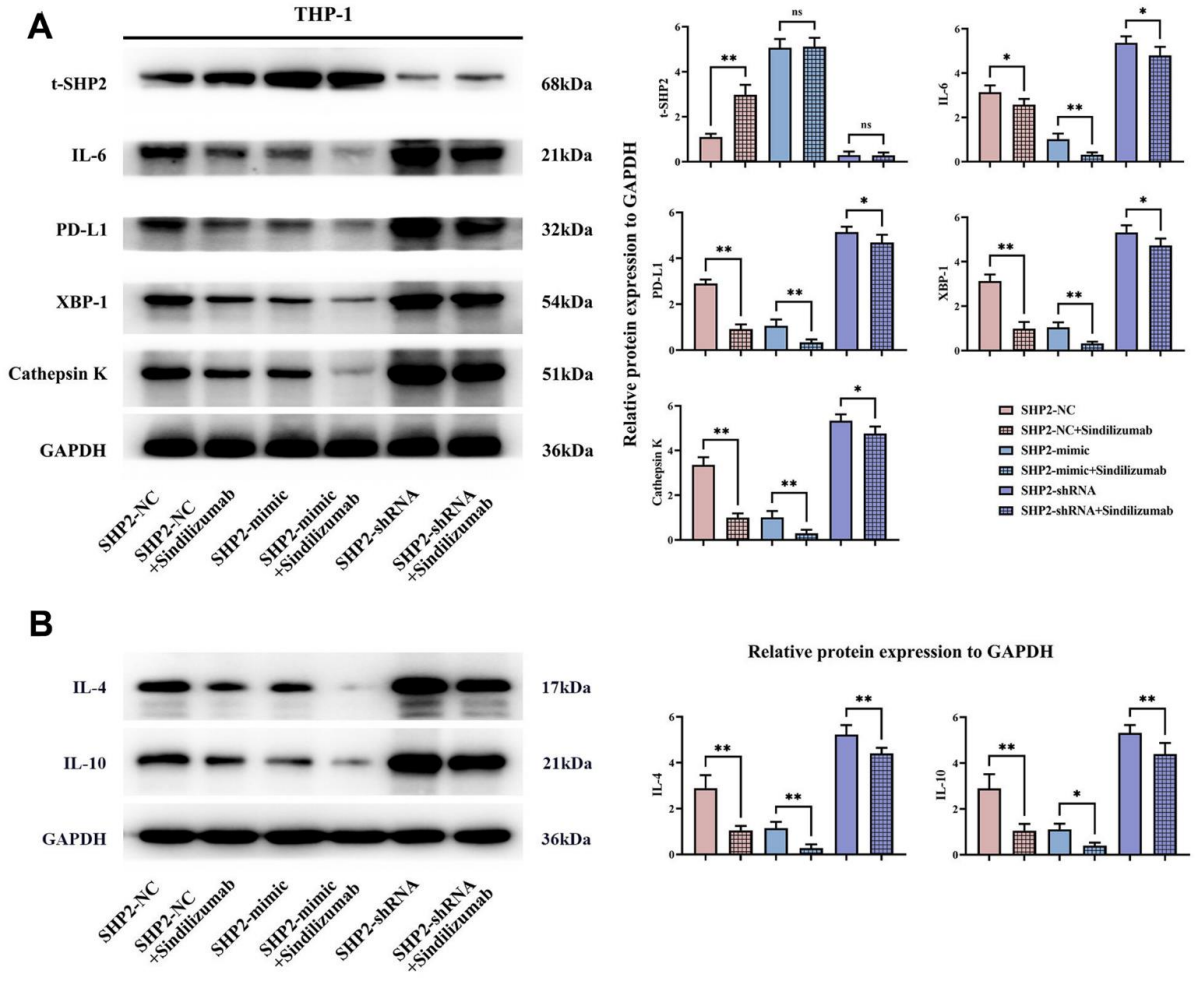
**Impact of PD-1 monoclonal antibodies in TAMs on the PD-1/IRE1α/SHP2 signaling pathway.** (**A**) Protein banding plots of t-SHP2, IL-6, PD-L1, XBP-1 and cathepsin K and relative protein expression statistics; (**B**) protein banding plots of IL-4 and IL-10 and relative protein expression statistics. GAPDH as a control protein. Data were expressed as mean±SD. *P<0.05; **P<0.01; nsP>0.05.

To explore the specific mechanism of the effect of Sindilizumab on cervical cancer, an *in vitro* experiment was established. Western blotting was used to detect the expression of related proteins in THP-1 cells. Compared to the SHP2-NC group, the SHP2-NC+Sindilizumab group exhibited significantly increased t-SHP2 expression and significantly decreased IL-6, IL-4, IL-10, PD-L1, XBP-1 and Cathepsin K expression; compared to the SHP2-mimic group, there were no significant changes in t-SHP2 expression in the SHP2-mimic+Sindilizumab group, but significant reductions in IL-6, IL-4, IL-10, PD-L1, XBP-1, and Cathepsin K; compared to the SHP2-shRNA group, there was no significant difference in t-SHP2 levels in the SHP2-shRNA+Sindilizumab group, but there were significant reductions in the levels of IL-6, IL-4, IL-10, PD-L1, XBP-1, and Cathepsin K (Figure 2).

### Sindilizumab in TAMs can regulate the PD-1/IRE1α/SHP2 signaling pathway to inhibit migration of cervical cancer cells

**Figure 3:**
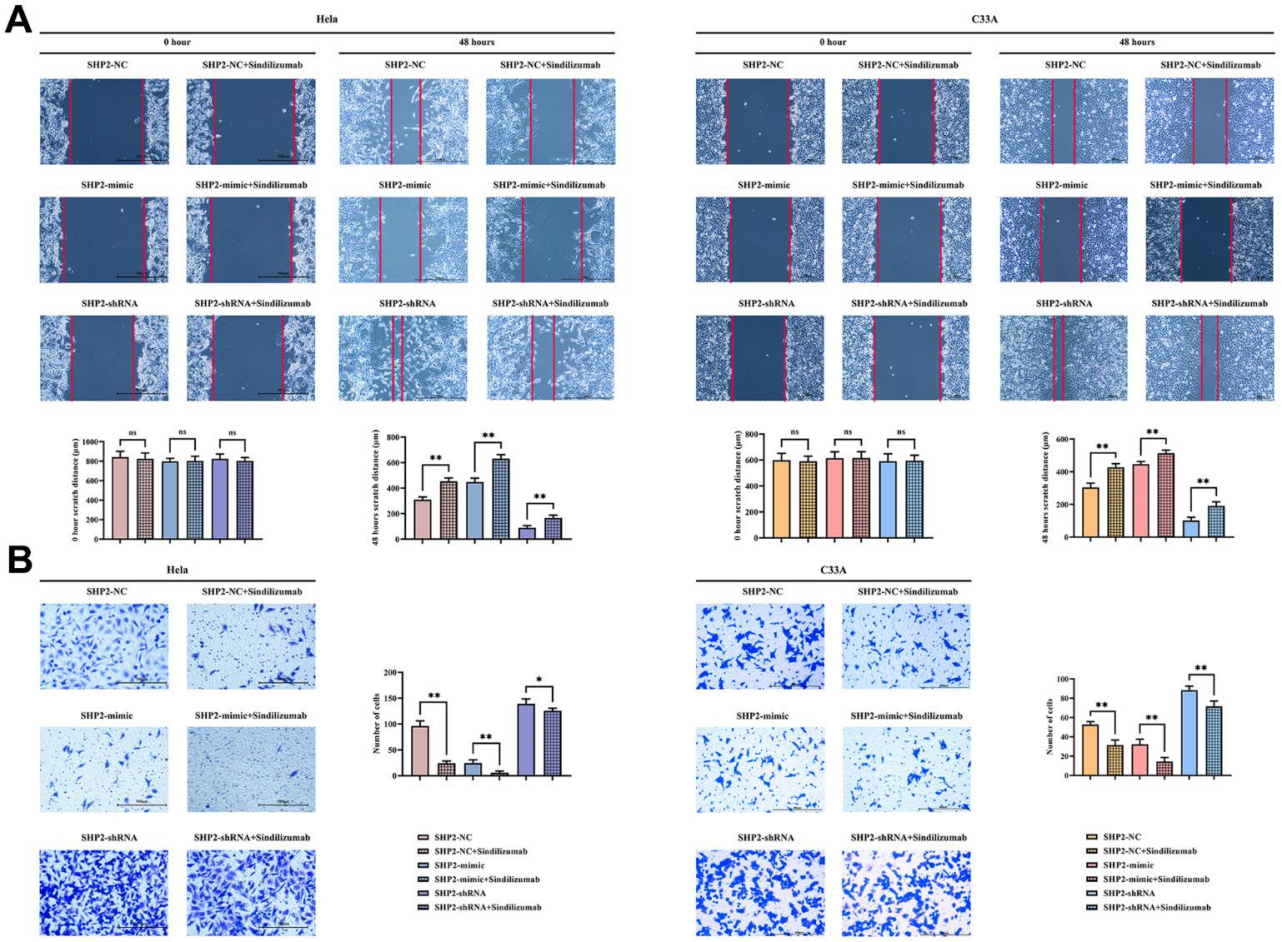
**Impact of PD-1 monoclonal antibodies in TAMs on the migration ability of cervical cancer cells.** (**A**) Wound healing results and cell spacing statistics of Hela cells and C33A cells; (**B**) Transwell migration assay results plot and statistics on the number of migrating cells for Hela cells and C33A cells. Data were expressed as mean±SD. *P<0.05; **P<0.01; nsP>0.05.

Wound healing assay and Transwell assay were used to detect the migration ability of Hela cells and C33A cells. The wound healing assay showed that at 48 hours, compared to the SHP2-NC group, the cell distance was significantly wider in the SHP2-NC+Sindilizumab group; for the SHP2-mimic group, the cell distance was significantly wider in the SHP2-mimic+Sindilizumab group; also, for the SHP2-shRNA group, there was a significant increase in the cell distance in the SHP2-shRNA+Sindilizumab group. Transwell results demonstrated that, compared to the SHP2-NC group, the number of migratory cells was significantly decreased in the SHP2-NC+Sindilizumab group; for the SHP2-mimic group, the cell migration count was significantly reduced in the SHP2-mimic+Sindilizumab group; likewise, for the SHP2-shRNA group, a significant reduction in the number of migratory cells was observed in the SHP2-shRNA+Sindilizumab group (Figure 3).

### Sindilizumab in TAMs can promote angiogenesis by regulating the SHP2/Src signaling pathway

**Figure 4:**
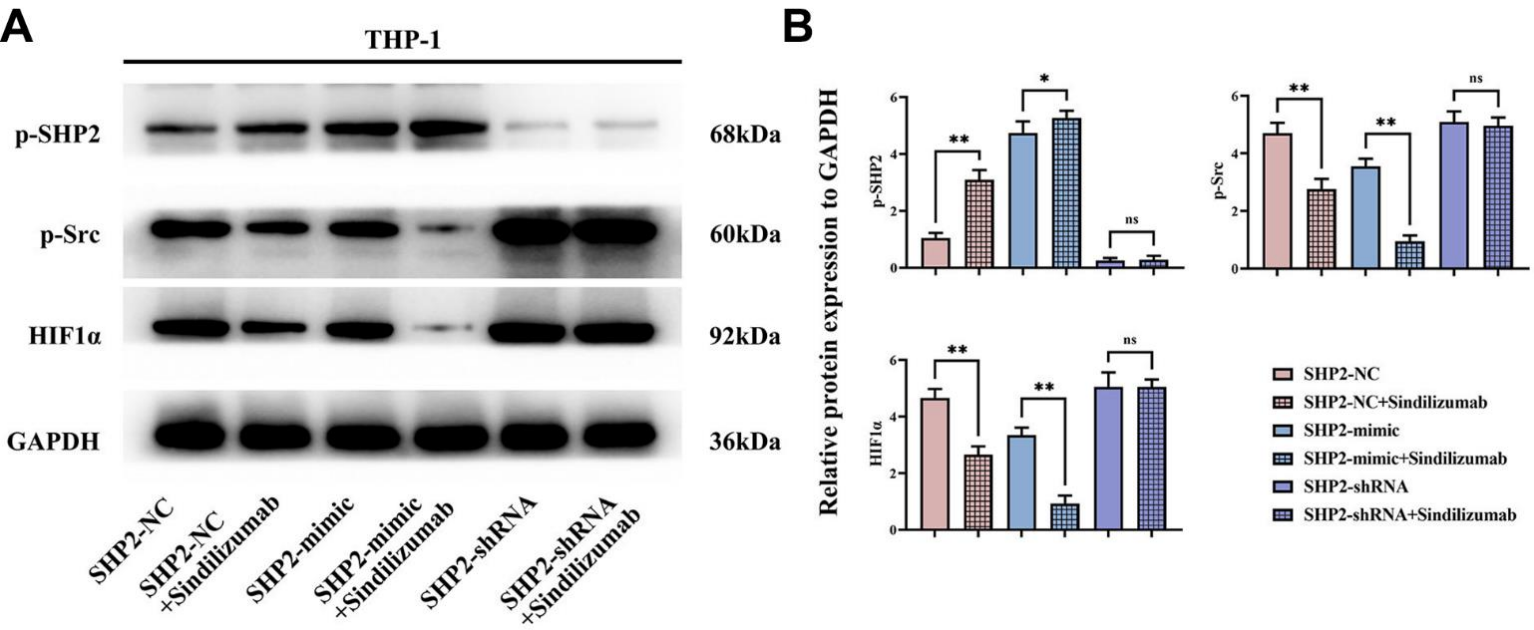
**Impact of PD-1 monoclonal antibodies in TAMs on the SHP2/Src signaling pathway.** (**A**) Western blot bands of p-SHP2, p-Src, and HIF1α; (**B**) statistical analysis of the relative protein expression levels of p-SHP2, p-Src, and HIF1α. GAPDH as a control protein. Data were expressed as mean±SD. *P<0.05; nsP>0.05.

Western blotting was used to detect the expression of related proteins in THP-1 cells. In the SHP2-NC group, relative protein expression levels of p-SHP2 were significantly increased after treatment with Sindilizumab, while the levels of p-Src and HIF1α were significantly decreased; compared to the SHP2-mimic group, the relative expression levels of p-SHP2 were significantly increased, and the levels of p-Src and HIF1α were significantly decreased in the SHP2-mimic+Sindilizumab group; compared to the SHP2- shRNA group, there was no significant difference in the relative protein expression levels of p-SHP2, p-Src, and HIF1α in the SHP2-shRNA+Sindilizumab group (Figure 4).

**Figure 5:**
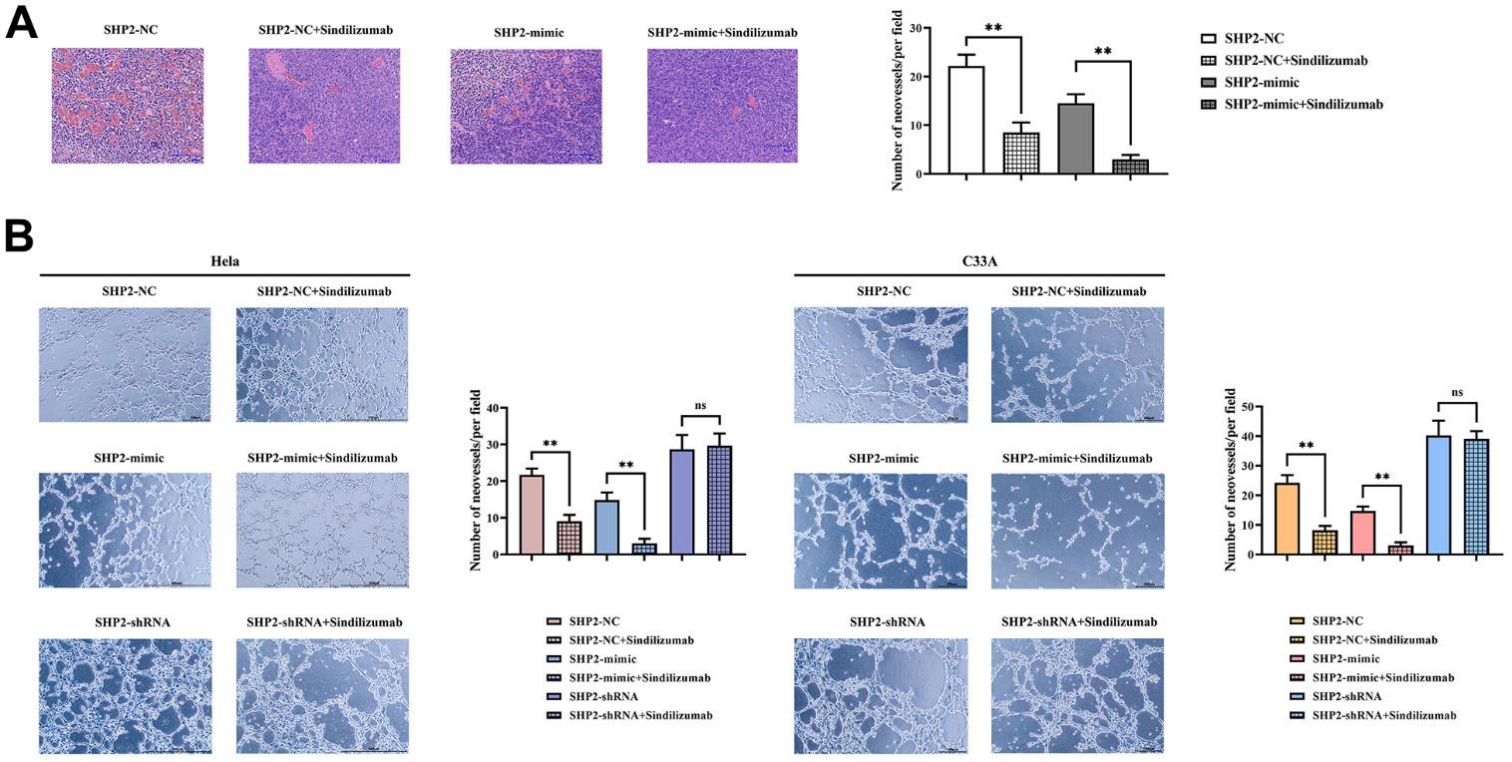
**Impact of PD-1 monoclonal antibodies in TAMs on the angiogenic ability of cervical cancer cells.** (**A**) H&E staining results graph and statistical graph of blood vessel numbers; (**B**) angiogenesis experimental results and vessel count statistics of Hela cells and C33A cells. Data were expressed as mean±SD. *P<0.05; nsP>0.05.

**Figure 6:**
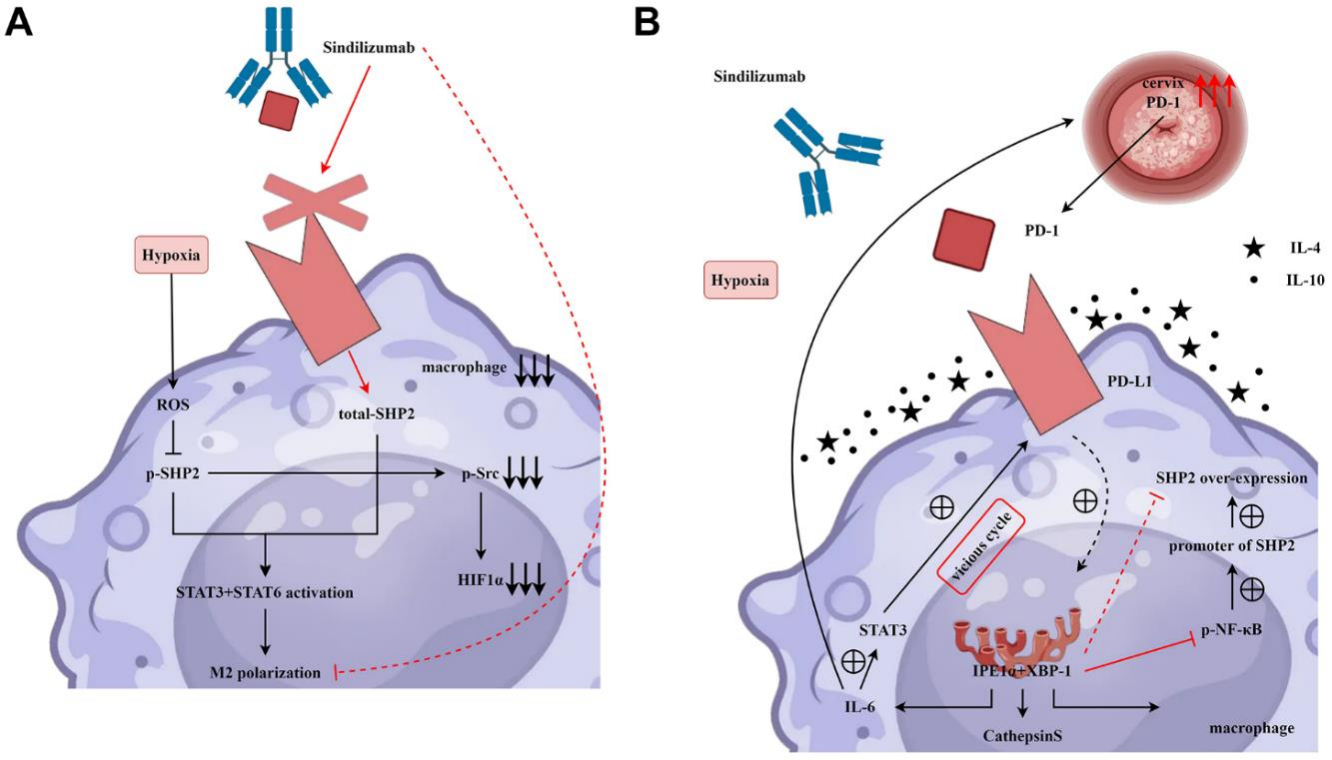
**PD-1 monoclonal antibodies in tumor-associated macrophages inhibit the migration and angiogenesis of cervical cancer cells by affecting the PD-1/IRE1α/SHP2/HIF1α signaling pathway, thereby suppressing the progression of cervical cancer.** (**A**) Sindilizumab inhibits M2 polarisation in tumour-associated macrophages by affecting the SHP2/STAT3/STAT6/HIF1α signalling pathway. (**B**) Sindilizumab inhibits migration and angiogenesis of cervical cancer cells by affecting the PD-1/IRE1α/SHP2/HIF1α signalling pathway.

HE staining results showed that after the addition of Sindilizumab, the number of blood vessels was significantly reduced in the SHP2-NC group and SHP2-mimic group compared to the SHP2-NC group, the blood vessel count was notably lower in the SHP2-mimic group; compared to the SHP2-NC+Sindilizumab group, the blood vessel count was significantly lower in the SHP2-mimic+Sindilizumab group. The results of angiogenesis experiments showed that in Hela cells and C33A cells, compared to the SHP2-NC group, the number of blood vessels was significantly decreased in the SHP2-NC+Sindilizumab group; compared to the SHP2-mimic group, the blood vessel count was significantly decreased in the SHP2-mimic+Sindilizumab group; there was no significant difference in blood vessel count between the SHP2-shRNA group and the SHP2-shRNA+Sindilizumab group (Figure 5). In conclusion, PD-1 monoclonal antibodies in tumor-associated macrophages can inhibit the migration and angiogenesis of cervical cancer cells by affecting the PD-1/IRE1α/SHP2/HIF1α signaling pathway, thereby inhibiting the progression of cervical cancer (Figure 6).

## DISCUSSION

Throughout the progression of cervical cancer, the neoplastic tissues frequently encounter hypoxia owing to accelerated proliferation and the development of defective vascular networks. This condition of oxygen deprivation within the tumor is identified as tumor tissue hypoxia. Hypoxia significantly influences cervical cancer by impacting tumor expansion, metastasis, and therapeutic responses. It is a detrimental prognostic indicator in cervical cancer, potentially prompting neoplastic cells to synthesize increased angiogenic factors, thereby amplifying angiogenesis and tumor proliferation. Furthermore, hypoxia can modify the metabolic routes of tumor cells, heightening their dependency on anaerobic glycolysis and enhancing their invasive and metastatic potential. Cervical cancer exhibits pronounced metastatic capabilities, which substantially complicates therapeutic interventions and patient outcomes [15, 16].

The tumor microenvironment constitutes the principal locus for the proliferation and differentiation of tumor cells, while the metastasis of these cells is linked to the secretion of inflammatory cytokines by Tumor-associated macrophages (TAMs). Within the context of cervical cancer, cytokines IL-4 and IL-10 play pivotal roles in modulating immune responses and tumor expansion. IL-4, a cytokine secreted by T cells, B cells, and macrophages, potentially facilitates tumor progression within this microenvironment. It can enhance the growth and dissemination of tumor cells and suppress the function of immune cells such as cytotoxic T cells, macrophages, and natural killer cells, thereby contributing to the tumor’s evasion of immune surveillance. Furthermore, IL-4 might influence the tumor cells’ resistance to radiotherapy and chemotherapy. Conversely, IL-10, an anti-inflammatory cytokine produced by assorted cells including T cells, B cells, and monocytes, may manifest anti-inflammatory properties within the tumor microenvironment and suppress the activity of immune cells, diminishing their cytotoxic impact on the tumor. IL-10 also supports immune tolerance within the tumor, thereby aiding its growth and spread [17–19].

In cervical cancer, the PD-1/PD-L1 axis and tumor-associated macrophages are instrumental within the tumor microenvironment. PD-1 is an immune checkpoint molecule prominently expressed on T cells and macrophages. Interaction between PD-1 and its ligand PD-L1 suppresses immune cell function, thus protecting cancer cells from immune attacks. Elevated PD-L1 expression allows tumor and other immune cells to evade immunological destruction. Therapeutic inhibition of the PD-1/PD-L1 pathway with PD-1 monoclonal antibodies reinvigorates immune cells’ ability to target cervical cancer cells, thus impeding cancer progression and enhancing SHP2 expression [20, 21]. This study found that using PD-1 monoclonal antibodies can inhibit the progression of cervical cancer and promote the expression of SHP2.

IL-6 likely plays an important role in the tumor microenvironment of cervical cancer. IL-6 is a cytokine that can enhance the development and progression of various cancers, including cervical cancer. It promotes tumor growth and metastasis, and regulates the functions of T cells, macrophages, and other immune cells, thus influencing the tumor's immune escape capabilities. IL-6 may also trigger the expression of PD-L1 through the activation of the STAT3 signaling pathway. PD-L1 expression is modulated by the endoplasmic reticulum stress signaling pathway and may be facilitated through transcription factors, protease activity, and other mechanisms, thereby augmenting PD-L1 levels. Additionally, PD-L1 may encourage the expression of endoplasmic reticulum stress proteins. Research indicates that XBP1 can directly or indirectly influence the expression of IL-6. One study suggests that under conditions of endoplasmic reticulum stress, activated XBP1 interacts with the promoter of IL-6, enhancing its transcription and expression. Furthermore, XBP1 can engage with other transcription factors such as NF-κB (nuclear factor kappa B) to coordinate the regulation of IL-6 gene expression and the expression of proteases, while NF-κB can inhibit the expression of SHP2 [22–25]. Therefore, in this study, TAMs with PD-1 monoclonal antibodies can promote the expression of t-SHP2, inhibiting the relative protein expression of IL-6, IL-4, IL-10, PD-L1, XBP-1, and cathepsin K. Moreover, TAMs with PD-1 monoclonal antibodies can inhibit the migration ability of cervical cancer cells.

SHP2 plays a pivotal regulatory role in numerous cellular signaling cascades, chiefly promoting the RAS-MAPK pathway. It functions via its SH2 domain, recognizing specific phosphorylated tyrosine residues while integrating cellular signals by dephosphorylating proteins, thus modulating SRC kinase activity or related signaling cascades. Src kinase, a part of the SRC family, can elevate HIF-1α activity, a key transcription factor activated under hypoxic conditions, thereby regulating genes central to adapting to hypoxia [26, 27]. Studies have shown that Src kinase can enhance the activity of HIF-1α in a variety of ways. First, Src kinase can affect the stability and activity of HIF-1α by regulating its phosphorylation state. Second, Src kinase may enhance the transcriptional activity of HIF-1α by promoting its nuclear translocation. In addition, Src kinase can also impact the function of HIF-1α by regulating its translation process or its interaction with other proteins, which in turn promotes angiogenesis, glycolysis, and other metabolic pathway regulations. In this study, TAMs with PD-1 monoclonal antibodies can promote the expression of p-SHP2, inhibiting the relative protein expression of p-Src and HIF1α. Moreover, TAMs with PD-1 monoclonal antibodies can inhibit the angiogenic ability of cervical cancer cells. However, this study needs to be followed up with relevant clinical trials to provide a stronger basis for the conclusions of this study.

In conclusion, PD-1 monoclonal antibodies in tumor-associated macrophages can inhibit the migration and angiogenesis of cervical cancer cells by affecting the PD-1/IRE1α/SHP2/HIF1α signaling pathway, thereby inhibiting the progression of cervical cancer. This provides new therapeutic targets for the treatment of cervical cancer.

## MATERIALS AND METHODS

### Cell culture and treatment

Human macrophages THP-1, and human cervical cancer cell lines Hela and C33A were purchased from Wuhan Puno Sai Biotechnology Co., Ltd. (China). All cells were cultured in Dulbecco's Modified Eagle Medium containing 10% fetal bovine serum, 100 U/mL penicillin, and 100 U/mL streptomycin. The plasmids for overexpressing SHP2, SHP2 shRNA and the corresponding negative control vectors were obtained from GenePharma (Shanghai, China). THP-1 cells were seeded in 6-well plates with 2 mL of antibiotic-free growth medium. The cells were allowed to attach until 70% confluence. The plasmids were transfected using Lipofectamine 2000 (Invitrogen, USA) following the manufacturer's instructions. The cells were incubated at 37° C (5% CO_2_) for 12 h, followed by replacement of the culture medium with medium containing FBS. 48 hours post-transfection, THP-1 cells were co-cultured indirectly with Hela and C33A cells, divided into SHP2-NC group, SHP2-mimic group, and SHP2-shRNA group. PD-1 monoclonal antibodies (Sindilizumab) were added to set up control groups. They were cultivated in 1% O_2_ conditions and supplemented with IL-4 and IL-10.

### Nude mouse tumor bearing experiment

25 six-week-old female nude mice weighing 20±1g were purchased from Henan Sike Best Life Technology Co., Ltd. (China). Mice were housed in a clean, sterile environment with a temperature maintained at 22° C and humidity at 60%, with a 12/12-hour light-dark cycle. After one week of acclimation feeding, the mice were randomly divided into four groups (n=6 per group). The cell suspensions of THP-1 and C33A cells from the SHP2-NC group, SHP2-mimic group, SHP2-NC+Sindilizumab group, and SHP2-mimic+Sindilizumab group were injected subcutaneously into the right flank of each mouse. 16 days post-inoculation, tumor size was measured every 3 days with a digital caliper (V=π/6×L×W×W, L being the longest diameter, W being the shortest diameter). Before euthanasia with 20% isoflurane gas, *in vivo* imaging was performed on all mice 4 weeks after the measurements began. The death of the mice was confirmed with cervical dislocation. The animal experiments in this study have been approved by the Medical Ethics Committee of the First Affiliated Hospital of Hebei North University, and the ethical batch number is K2021068.

### HE staining

Tumor tissues were cut into blocks measuring 1.0cm×1.0cm×0.5cm, fixed in neutral formalin solution for 3 days, decalcified in 30% formic acid solution for 14 days, and then conventionally dehydrated with ethanol. Samples were embedded in paraffin and cut into 5 μm thick sections. Tumor samples were stained with Harris hematoxylin solution for 5 minutes after deparaffinization and rehydration. After washing with 0.5% acid-alcohol for 10 seconds, the sections were stained in eosin for 40 seconds. After dehydration, clearing, and mounting with neutral-resin, the samples were observed under a microscope.

### Wound healing assay

Logarithmically growing Hela and C33A cells were seeded into 6-well plates, and after cells reached confluence, a vertical scratch was made using a 20 μl pipette tip. The cells were then washed with phosphate-buffered saline (PBS) to remove the detached cells, and the cancer cells were cultured in serum-free medium. Photographs were taken at 0 and 48 hours under an optical microscope. Cell distances were calculated using ImageJ software.

### Transwell assay

According to the manufacturer's instructions, a Transwell chamber (Corning, USA) was used to assess the migration of Hela and C33A cells *in vitro*. Cell suspensions of Hela and C33A cells (400 μl, 3×104 cells/well) were seeded in the upper chamber, and migration assays were carried out without matrix gel coating. A 700 μl medium containing 10% FBS was added to the lower chamber and the cells that migrated to the basement membrane were fixed with 4% paraformaldehyde and stained with crystal violet. The cells were observed at 100× magnification under an IX73 microscope (Olympus, Tokyo, Japan) and photographed with a DP80 imaging system. The number of migrating cells was counted.

### Angiogenesis assay

Differentiation of HUVECs was checked via tube formation assay on Matrigel. Matrigel was thawed overnight on ice, spread uniformly at 50 μL/well in a 96-well plate, and polymerized at 37° C for one hour. HUVECs (6×104 cells/well) were seeded onto the Matrigel layer and cultured in DMEM supplemented with 1% FBS at 37° C. After 8 hours, tube formation was observed and captured with a phase-contrast microscope. Total tube length per well was determined by computer-assisted image analysis using Image-Pro Plus software.

### Western blotting

After digesting with trypsin and washing with PBS, cells or tissues were lysed with RIPA lysis buffer, and total protein of cells was prepared. Protein concentration was measured using the BCA method. Samples were mixed with loading buffer and boiled in a metal bath for 10 minutes. Proteins (20 μg/well) were separated by electrophoresis on a polyacrylamide gel and then transferred onto PVDF membranes. The membranes were blocked with 5% skim milk at room temperature for 2 hours, and incubated with primary antibodies at 4° C overnight. The next day, after TBST washes, the membranes were incubated with HRP-conjugated secondary antibodies at room temperature for 2 hours. After additional washes with TBST, an appropriate amount of ECL luminescent liquid was dripped on them. Exposures were carried out in a gel-imaging system and grayscale values were analyzed.

### Statistical analysis

Data and images were analyzed using Prism 9.0 software. All experiments were counted three times. Measurement data were expressed as x±s. For data that followed a normal distribution, t-tests were used when P<0.05, indicating statistical significance.

### Data availability statement

All data generated or analyzed during this study are included in this published article (and its supplemental information document).
